# Integrated Bioinformatics Identification and Multi-Dimensional Characterization of Diagnostic Hub Genes in Colorectal Cancer

**DOI:** 10.3390/cimb48090916

**Published:** 2026-09-08

**Authors:** Zeynep Mutlu Coskun, Muhammet Coskun, Saime Sezer Sondas

**Affiliations:** 1 Department of Medical Biology, Faculty of Medicine, Malatya Turgut Ozal University, Malatya 44210, Türkiye; 2 Department of Biomechanics, Institute of Health Sciences, Dokuz Eylül University, Izmir 35330, Türkiye

**Keywords:** colorectal cancer, bioinformatics, diagnostic biomarker, AQP8, tumour immune microenvironment, stemness

## Abstract

Colorectal cancer (CRC) motivates the investigation of complementary molecular markers, although tissue-transcriptomic discrimination does not by itself establish early-detection performance. We identified differentially expressed genes in 98 paired CRC and adjacent-normal samples from GSE44076 using gene-level limma analysis (|log2FC| > 1; Benjamini–Hochberg-adjusted *p* < 0.05), screened candidates through a STRING protein–protein interaction network, and evaluated diagnostic performance with independent validation in GSE9348. Functional characterization included MSigDB KEGG_LEGACY over-representation analysis and preranked GSEA, immune-marker correlations, mRNA stemness index analysis, exploratory topology-filtered cysteine assessment, exploratory TCGA-COAD prognostic modelling, and literature-curated single-cell contextualization. Seven candidates—AQP8, CA7, GUCA2A, GUCA2B, BEST4, TMIGD1, and OTOP2—showed AUC values of 0.986–0.995 in the discovery cohort; five genes showed AUC values of 0.999–1.000 in the retrospective GSE9348 tissue cohort. AQP8 and GUCA2B showed weak nominal inverse correlations with mRNAsi that did not remain significant after correction across six tests (BH-FDR = 0.078), and the genes were predominantly associated with differentiated colonic epithelial lineages. The exploratory prognostic model was non-significant. These results identify seven tissue-transcriptomic candidates with strong dataset-specific discriminative performance and provide a multidimensional biological framework warranting experimental validation and prospective clinical evaluation.

## 1. Introduction

Colorectal cancer (CRC) ranks as the third most frequently diagnosed malignancy and the second leading cause of cancer-related mortality globally, with 1.93 million new cases and 935,000 deaths annually [1]. The five-year survival rate exceeds 90% when CRC is detected at a localized stage but drops below 15% in advanced metastatic disease, underscoring the critical importance of early diagnosis [2]. Despite advances in colonoscopy-based screening, participation rates remain suboptimal in many populations, and there is a continued clinical need for molecular biomarkers that could support tissue-based investigation and, after dedicated assay development, be evaluated for potential future non-invasive applications [3].

High-throughput microarray and RNA-sequencing technologies have enabled genome-wide identification of differentially expressed genes (DEGs) in CRC, revealing thousands of transcriptomic alterations associated with malignant transformation [4]. While CRC-focused bioinformatics studies have identified diagnostic and prognostic gene candidates from public repositories such as GEO and TCGA [5], and integrative transcriptomic frameworks have provided further biological insights through regulatory-network and machine-learning analyses [6], integrated multi-dimensional characterization spanning immune-marker co-expression, mRNA stemness index, topology-filtered cysteine prioritization, and cell-type-specific expression may help organize candidate-gene evidence within a single analytical framework. A comprehensive understanding of hub gene biology across these dimensions is essential for prioritizing candidates for downstream experimental validation and potential clinical translation.

The tumour immune microenvironment plays a central role in CRC prognosis and treatment response, particularly in the era of immunotherapy [7]. Cancer stemness, as quantified by the mRNA stemness index (mRNAsi), reflects the degree of oncogenic dedifferentiation and has been linked to therapy resistance and poor outcomes [8]. Post-translational modifications such as S-palmitoylation can regulate protein membrane localization and trafficking [9]. In the present study, immune-marker correlations, mRNAsi associations, and topology-filtered cysteine positions were evaluated only as exploratory, hypothesis-generating dimensions and do not establish immune infiltration, stemness mechanisms, or protein modification.

This study aimed to prioritize tissue-transcriptomic candidate genes in CRC through integrated bioinformatics analysis using discovery and independent retrospective cohorts, and to provide multi-dimensional characterization spanning pathway enrichment, immune-marker correlations, mRNA stemness index association, exploratory protein-topology assessment, prognostic modelling, and literature-curated single-cell context. We examined whether transcriptomically prioritized candidates showed reproducible dataset-specific tissue discrimination and biologically coherent, hypothesis-generating patterns across these analytical dimensions.

## 2. Materials and Methods

### 2.1. Data Sources and Preprocessing

Gene expression profiles were obtained from the NCBI Gene Expression Omnibus (GEO) (Supplementary Table S1). The discovery cohort was GSE44076 (Affymetrix HG-U219 platform; 98 primary CRC tissues and 98 paired adjacent normal colon mucosa specimens from Spanish patients) [10]. The independent validation cohort was GSE9348 (Affymetrix HG-U133 Plus 2.0; 70 primary CRC and 12 normal colon tissues) [11]. TCGA-COAD RNA-Seq expression data (514 samples: 41 normal mucosa and 473 tumour; RSEM-normalized counts) were downloaded from UCSC Xena (https://xena.ucsc.edu, accessed on 9 August 2026). Of these, 435 tumour samples had available follow-up data and were used for survival analyses; 274 had complete expression and clinical records and were used for the elastic-net prognostic model and mRNAsi correlation analyses. All microarray data were processed with RMA normalization and log_2_ transformation. Batch effects were assessed visually; no cross-platform normalization was required as cohorts were analyzed independently.

### 2.2. Differential Expression and Candidate Gene Identification

GSE44076 was profiled on the Affymetrix Human Genome U219 Array (GPL13667). Probe sets were mapped to official HGNC gene symbols; where multiple probe sets mapped to the same gene, the probe set with the highest mean expression across all 246 GSE44076 samples was retained, yielding 20,033 unique gene-level representatives. This selection was performed without reference to tumour or normal sample labels to avoid data leakage. The resulting gene-level matrix was then used to fit a paired limma model to the 98 CRC tumour and 98 matched adjacent-normal samples. DEGs were identified using thresholds of |log_2_FC| > 1 and Benjamini–Hochberg-adjusted *p* < 0.05. Differentially expressed genes were submitted to the STRING database (version 11.5; interaction confidence threshold ≥ 0.4) for PPI network construction [12]. After network construction, connected nodes were ranked by degree, and the 50 highest-degree genes were used as the operational candidate-screening list. Network membership and degree ranking were used only for screening and were not considered independent proof of hub status. BEST4 ranked 51st and was therefore not formally included in this list; however, it was retained as an exploratory near-threshold candidate because it lay immediately below the arbitrary cutoff, showed strong differential expression and diagnostic discrimination, and represented a biologically relevant marker of differentiated BEST4-positive colonic epithelial cells.

### 2.3. Functional Enrichment

Over-representation analysis was performed separately for the upregulated and downregulated differentially expressed genes using the MSigDB KEGG_LEGACY gene-set collection (CP:KEGG_LEGACY, Homo sapiens; msigdbr version 26.1.0). Gene symbols were mapped to unique NCBI Entrez Gene identifiers, and all mapped genes tested in the paired gene-level limma analysis were used as the background universe. Pathway enrichment was evaluated using a one-sided hypergeometric test, followed by Benjamini–Hochberg correction for multiple testing. Pathways with a BH-adjusted *p* value ≤ 0.05 were considered significant. Preranked gene set enrichment analysis was performed using a custom Python implementation [13] and the MSigDB KEGG_LEGACY collection. The ranked vector comprised 16,233 unique Entrez Gene identifiers ordered by the moderated t-statistic obtained from the paired gene-level limma analysis. Gene sets containing 10–500 genes represented in the ranked vector were retained. Enrichment scores were calculated using a weighted Kolmogorov–Smirnov-like running-sum statistic with a weighting exponent of 1. Competitive gene-set null distributions were generated using 10,000 random gene sets of matched size while retaining the ranked statistic vector unchanged. Continuity-corrected empirical *p* values were calculated as (k + 1)/(N_same-sign_ + 1), and Benjamini–Hochberg correction was applied globally across all 182 tested pathways. The random seed was 42. Positive normalized enrichment scores indicated enrichment in CRC tumour, whereas negative normalized enrichment scores indicated enrichment in matched adjacent-normal tissue. This custom implementation was not considered numerically equivalent to fgsea or the GSEA desktop application.

### 2.4. Diagnostic Evaluation

ROC curve analysis was performed using the pROC (1.19.0.1) package in both discovery and validation cohorts separately. AUC values with 95% confidence intervals and exploratory sensitivity and specificity at cohort-specific Youden index cutoffs were calculated for each candidate gene. Because these operating points were re-estimated within each cohort and no discovery-derived cutoff was transported across platforms, they are not interpreted as validation of a locked clinical threshold. Cross-platform discrimination was assessed in GSE9348 after log_2_ transformation of the available expression intensities.

### 2.5. Survival Analysis

Overall survival was assessed using the GEPIA2 TCGA-COAD survival module (median expression split, log-rank test) [14]. Additionally, direct TCGA-COAD survival analysis was performed using both median and quartile splits on 435 patients with available follow-up data. An exploratory six-gene prognostic risk model (AQP8, CA7, GUCA2A, GUCA2B, BEST4, and TMIGD1) was constructed using elastic net-penalized Cox proportional hazards regression (L1 ratio = 0.5, alpha determined by 10-fold cross-validation, minimum deviance criterion) on 274 TCGA-COAD patients with complete expression and survival data.

### 2.6. Immune Microenvironment Analysis

Spearman correlations were calculated between six candidate genes (AQP8, CA7, GUCA2A, GUCA2B, BEST4, and TMIGD1) and selected immune-marker genes (CD8A, CD4, FOXP3, CD19, NCAM1, CD68, CD274, IDO1, and CTLA4) in GSE44076 CRC tumour samples. Unlike deconvolution frameworks such as CIBERSORT [15], these markers were used only as expression proxies; the analysis did not directly estimate immune-cell abundance or infiltration. OTOP2 was not evaluated in this exploratory immune-marker analysis, and no immune-association inference is made for OTOP2.

### 2.7. mRNAsi Analysis

OCLR-derived mRNA stemness index (mRNAsi) scores were obtained from UCSC Xena PanCanAtlas (Malta et al. 2018) [8]. Spearman correlations were calculated between six candidate genes (AQP8, CA7, GUCA2A, GUCA2B, BEST4, and TMIGD1) and mRNAsi for 274 matched TCGA-COAD samples. OTOP2 was not evaluated in this secondary mRNAsi analysis.

### 2.8. S-Palmitoylation Assessment

Topology-filtered candidate cysteines were prioritized from UniProt-annotated protein topology by applying an exploratory ±5-residue juxtamembrane cytoplasmic rule [9]. This positional heuristic is not a validated S-palmitoylation predictor and does not demonstrate modification.

### 2.9. Single-Cell Contextualization

Hub gene cell-type expression patterns were curated from published colorectal and colonic single-cell RNA-seq atlases [16,17,18]. Qualitative expression levels were assigned based on reported marker gene patterns.

### 2.10. Statistical Analysis

Statistical analyses were performed using R (version 4.5.3) and Python 3.9. Differential-expression and enrichment analyses were conducted using the R packages described above, whereas survival and prognostic modelling analyses were performed using the specified Python packages (scipy, lifelines, and scikit-learn).

## 3. Results

### 3.1. Differential Expression and Candidate Gene Selection

**Figure 1:**
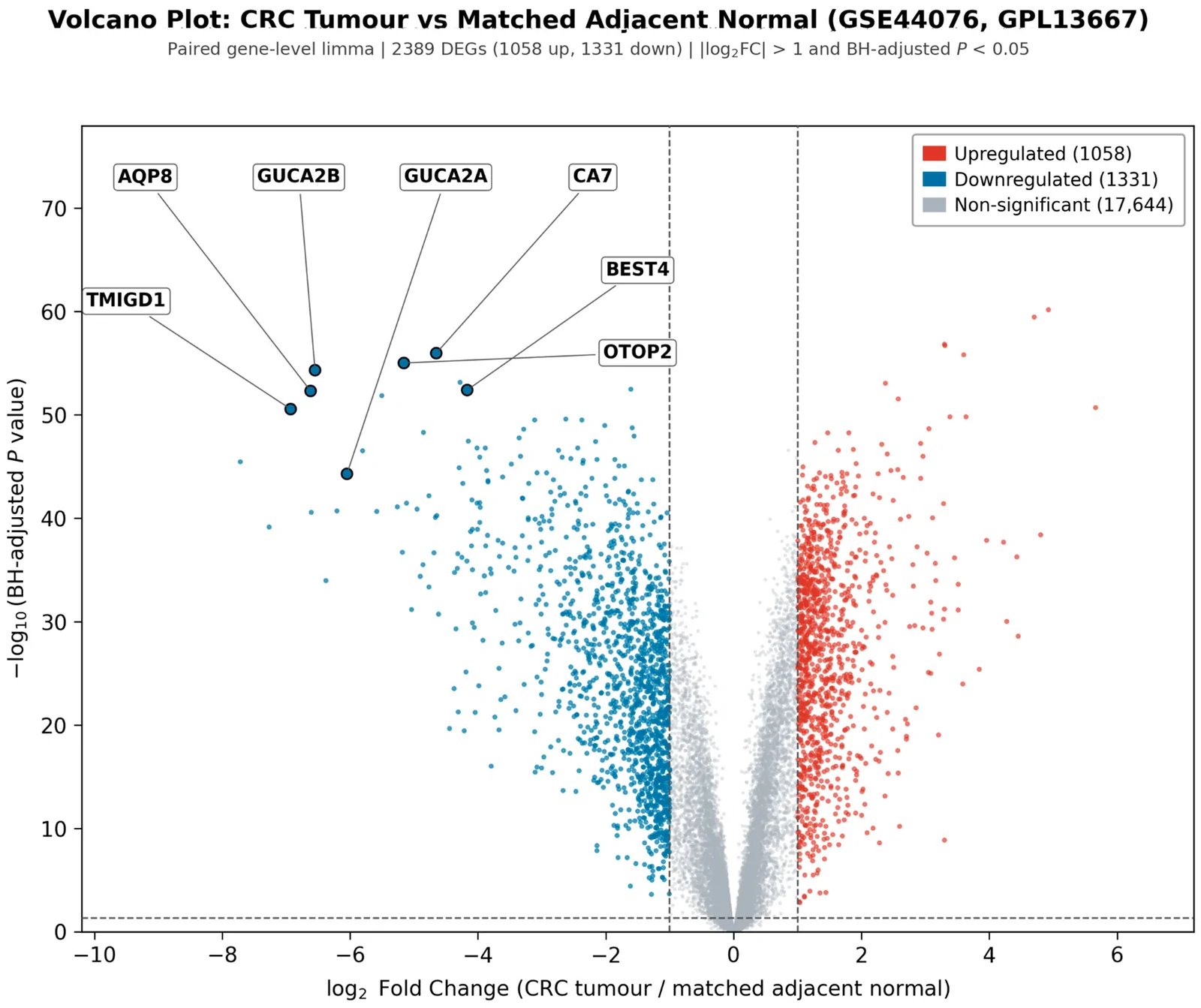
Volcano plot of paired gene-level differential expression in GSE44076. The analysis compared 98 CRC tumour samples with 98 matched adjacent-normal samples and identified 2389 DEGs (1058 upregulated and 1331 downregulated) using |log2FC| > 1 and BH-adjusted *p* < 0.05. Red indicates upregulated genes, blue indicates downregulated genes, and grey indicates non-significant genes. Dashed lines indicate the significance thresholds: |log_2_FC| = 1 (vertical) and BH-adjusted *p* = 0.05 (horizontal).

The paired gene-level analysis identified 2389 differentially expressed genes, including 1058 upregulated and 1331 downregulated genes, using |log_2_FC| > 1 and BH-adjusted *p* < 0.05 (Figure 1; Supplementary Table S2). Six of the seven candidate genes were present in the top-50 degree-ranked PPI candidate list (Supplementary Figure S1): TMIGD1 (rank 3), AQP8 (rank 4), GUCA2B (rank 6), GUCA2A (rank 9), OTOP2 (rank 16), and CA7 (rank 30). BEST4 ranked 51st and was not part of the formal top-50 degree-ranked list. It was nevertheless retained as an exploratory near-threshold candidate based on its proximity to the operational cutoff, strong differential expression, high discovery-cohort diagnostic accuracy (AUC = 0.986), and established relevance to differentiated colonic epithelial identity. This was an exploratory, post hoc prioritization rather than a prespecified classifier-development rule. The seven genes retained for subsequent diagnostic evaluation were AQP8, CA7, GUCA2A, GUCA2B, BEST4, TMIGD1, and OTOP2.

### 3.2. Pathway Enrichment

**Figure 2:**
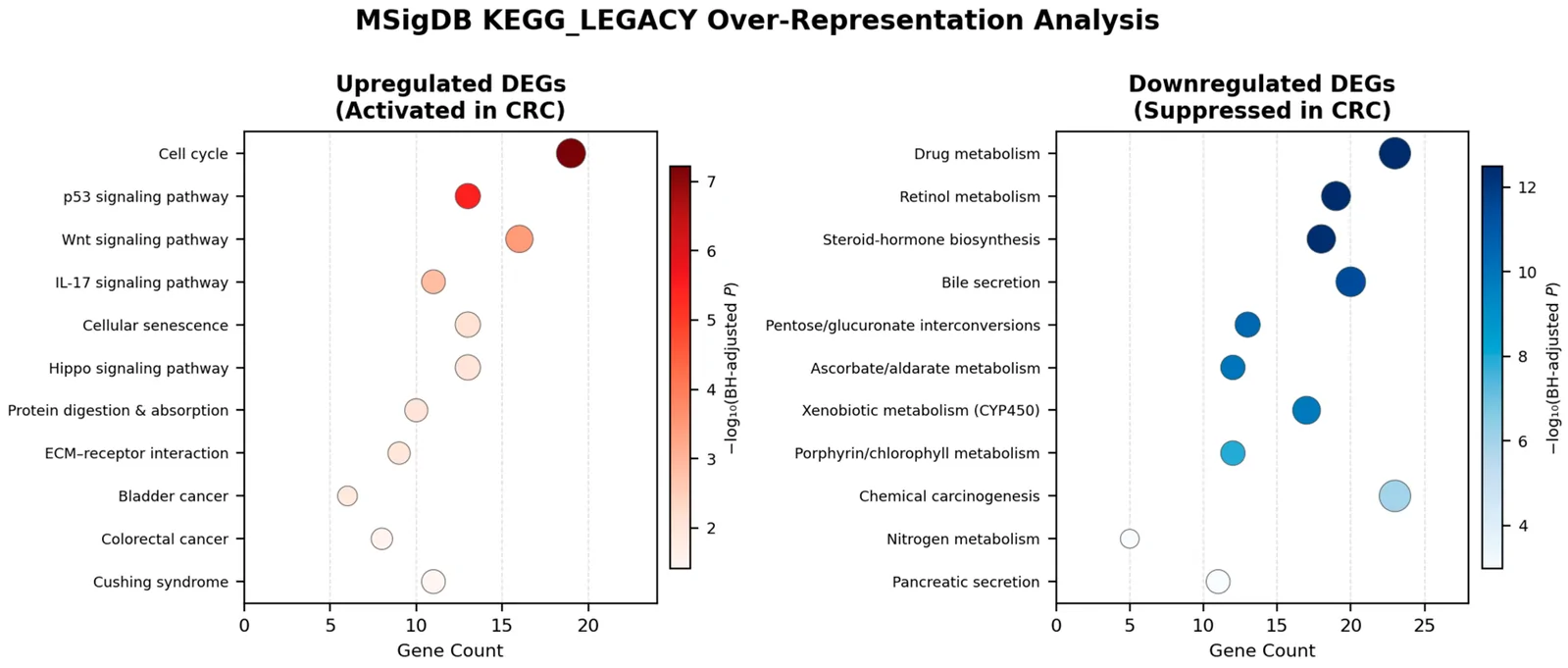
MSigDB KEGG_LEGACY over-representation analysis of differentially expressed genes. The upregulated and downregulated gene sets were analysed separately using a one-sided hypergeometric test with Benjamini–Hochberg correction. Circle size is proportional to the number of DEGs annotated to the pathway (gene count), the same quantity shown on the x-axis. Colour represents −log10 of the BH-adjusted *p* value.

MSigDB KEGG_LEGACY over-representation analysis revealed distinct pathway signatures (Figure 2; Supplementary Tables S3 and S4). Among the most significant pathways, upregulated DEGs were enriched in cell-cycle and DNA-replication programs, whereas downregulated DEGs were enriched in fatty-acid and drug-metabolism pathways. Preranked GSEA tested 182 MSigDB KEGG_LEGACY pathways. Of these, 115 pathways met the global BH-FDR threshold of 0.25, comprising 27 pathways with positive NES and 88 pathways with negative NES. Sixty-six pathways met the more stringent global BH-FDR threshold of 0.05, comprising 17 pathways with positive NES and 49 pathways with negative NES. Supplementary Figure S2 and Supplementary Table S5 present the 20 pathways with the largest absolute NES among pathways with global BH-FDR ≤ 0.25.

### 3.3. Diagnostic Performance

**Figure 3:**
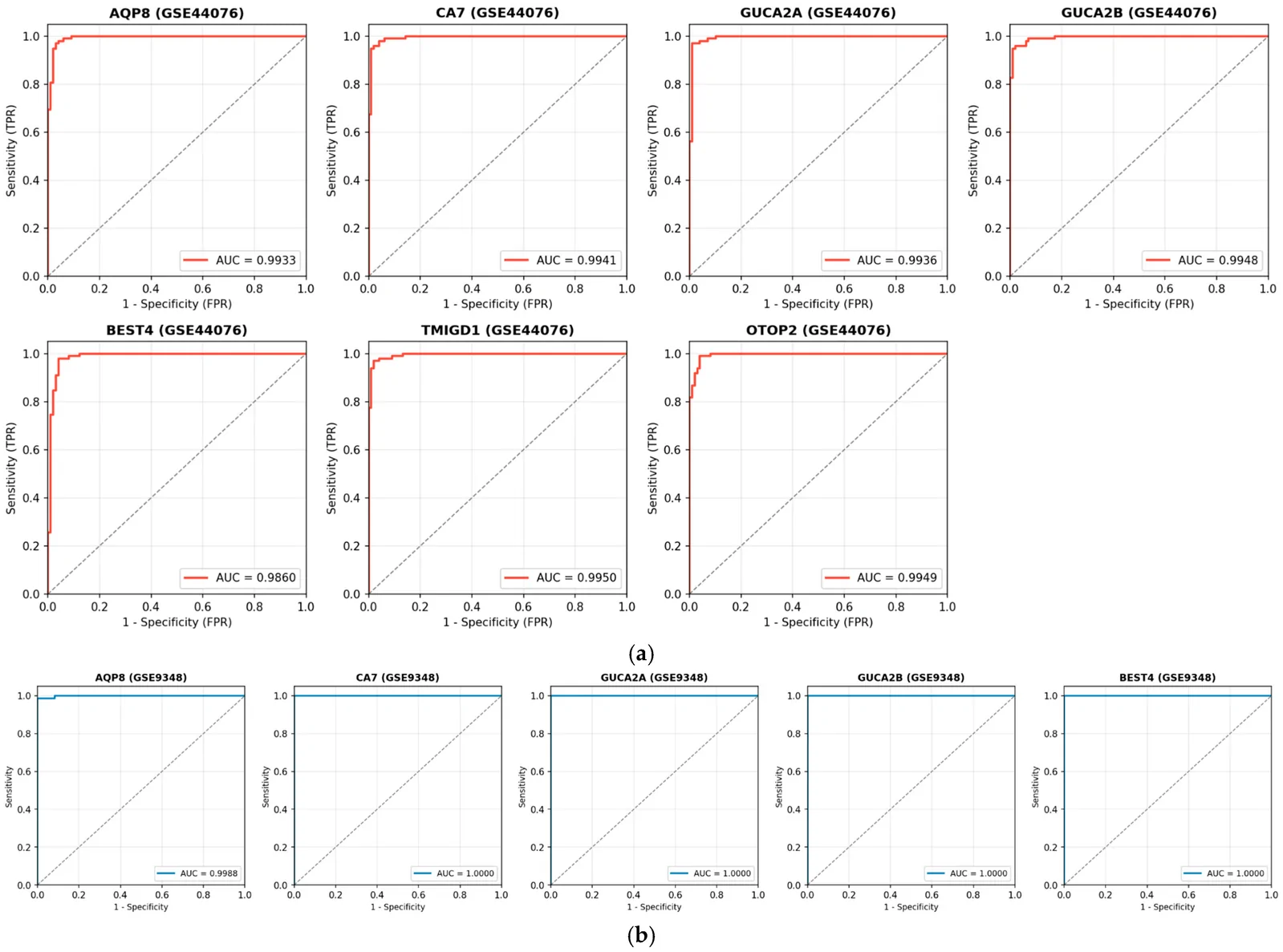
ROC curves for diagnostic performance. (**a**) Discovery cohort GSE44076 (98 CRC vs. 98 matched adjacent-normal samples); (**b**) validation cohort GSE9348 (70 CRC vs. 12 normal samples). AUC values are shown for each candidate gene.

All seven discovery-stage candidate genes showed strong dataset-specific tissue discrimination (Figure 3; Supplementary Table S6). In the discovery cohort (GSE44076), AUC values ranged from 0.986 (BEST4) to 0.995 (TMIGD1, GUCA2B, OTOP2). In the independent validation cohort (GSE9348), AUC values reached 0.999–1.000 for all five validated genes (AQP8, GUCA2A, GUCA2B, CA7, BEST4), showing near-perfect discrimination between CRC and normal tissue in this selected retrospective cohort. TMIGD1 was not represented on the GPL570 platform in GSE9348; OTOP2 showed non-significant change (+0.50, *p* = 0.094), suggesting dataset-specific variation. Cross-cohort validation confirmed statistically significant downregulation of AQP8 (log_2_FC = −8.40), GUCA2B (−8.91), GUCA2A (−6.66), and CA7 (−3.76). BEST4 showed concordant downregulation (log_2_FC = −4.76), although probe-level *p* values were not available in the derived validation summary; OTOP2 showed a non-significant change (log_2_FC = +0.50, *p* = 0.094) (Supplementary Table S7).

### 3.4. Survival Analysis

GEPIA2 analysis of TCGA-COAD data revealed that high AQP8 expression was significantly associated with improved overall survival (logrank *p* = 0.022, HR = 0.57; Supplementary Figure S3A; Supplementary Table S8), representing a nominal exploratory survival association. Other hub genes (GUCA2A, CA7, GUCA2B, BEST4) did not reach statistical significance (Supplementary Figure S3B–E). TMIGD1 was evaluated in GEPIA2 but did not reach statistical significance (log-rank *p* = 0.410) and is reported in Supplementary Table S8 only; no Kaplan–Meier survival panel is shown for this gene. Direct TCGA-COAD survival analysis showed a significant association using the median split (log-rank *p* = 0.019), whereas the quartile-based comparison did not reach statistical significance (*p* = 0.289), indicating sensitivity to the expression cut-off strategy (Supplementary Figure S4). The exploratory elastic net-penalized prognostic model assigned non-zero coefficients to all six genes included in the model and stratified patients into high-risk (27.0% mortality) and low-risk (21.2% mortality) groups; however, the difference did not achieve statistical significance (log-rank *p* = 0.639, cross-validated AUC = 0.503; Supplementary Figure S5; Supplementary Table S9). This null exploratory model did not establish prognostic utility in this cohort; it does not demonstrate absence of prognostic effects or prove a specific role in tumour initiation rather than progression.

### 3.5. Immune Microenvironment Associations

**Figure 4:**
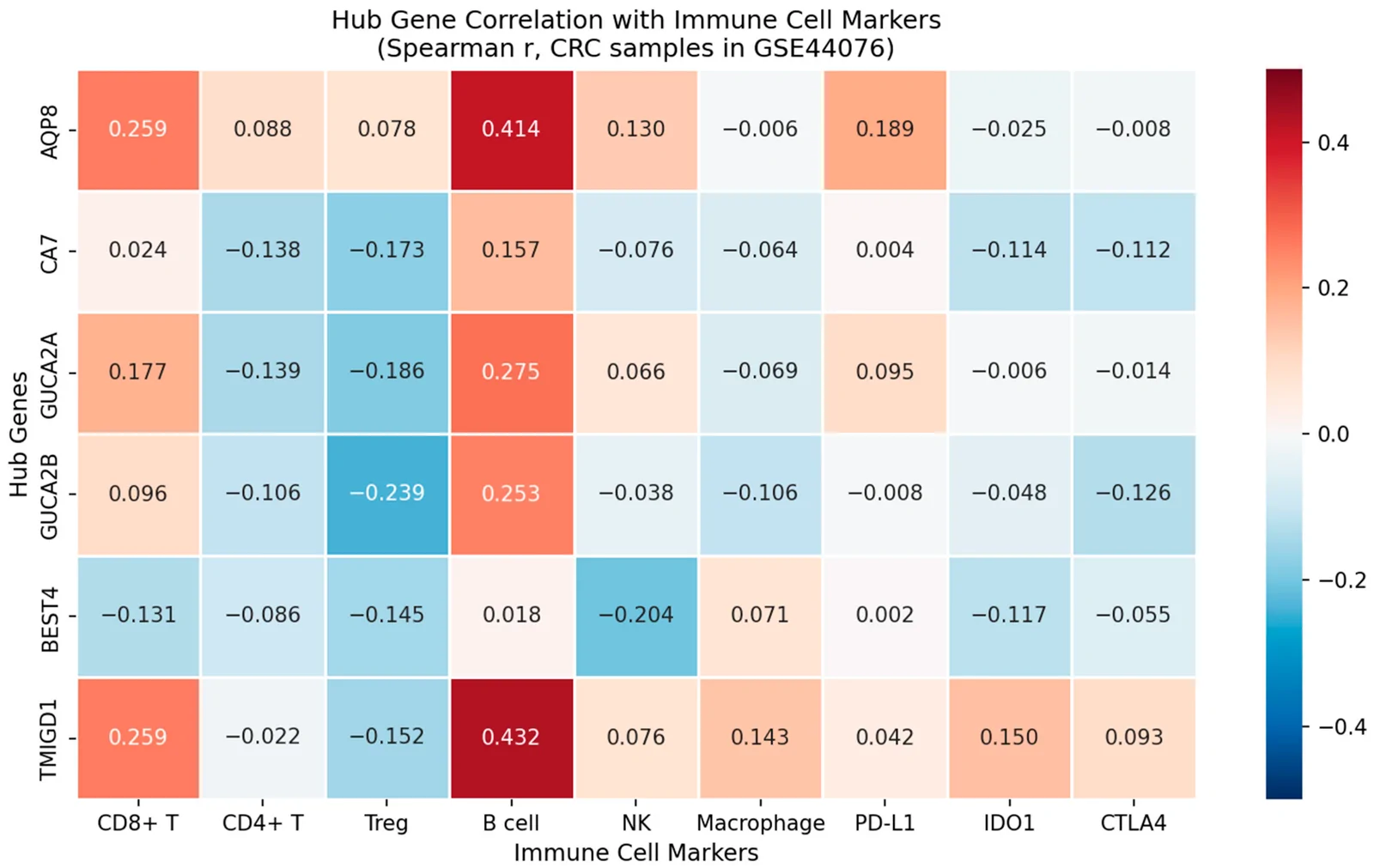
Immune microenvironment correlation heatmap. Spearman correlation coefficients between the six evaluated candidate genes and selected immune-marker genes in CRC tumours from GSE44076. OTOP2 was not evaluated in this analysis.

Correlation analysis for the six evaluated candidate genes and immune-marker genes revealed exploratory co-expression associations (Figure 4; Supplementary Table S10). Exploratory gene-specific correlations were observed between hub-gene expression and selected immune-marker genes in bulk tumour microarray expression data. AQP8 and TMIGD1 showed moderate positive correlations with CD19 (r = 0.414 and r = 0.432, respectively), whereas several other hub genes showed weak inverse correlations with FOXP3. These associations do not directly measure immune-cell abundance, infiltration, or immune activity. OTOP2 was not evaluated in this analysis.

### 3.6. Stemness Correlation

Correlation analysis using Malta et al. OCLR-derived mRNAsi scores (*n* = 274 TCGA-COAD patients) revealed weak nominal negative correlations for AQP8 (ρ = −0.142, nominal *p* = 0.019, BH-adjusted *p* = 0.078) and GUCA2B (ρ = −0.135, nominal *p* = 0.026, BH-adjusted *p* = 0.078) (Supplementary Figure S6; Supplementary Table S11). The remaining evaluated genes showed non-significant negative trends: CA7 (ρ = −0.001, *p* = 0.986), GUCA2A (ρ = −0.074, *p* = 0.223), BEST4 (ρ = −0.057, *p* = 0.345), and TMIGD1 (ρ = −0.042, *p* = 0.491). Among the six evaluated genes, all correlations were directionally negative, but none reached BH-FDR < 0.05 across the six tested genes. OTOP2 was not evaluated in this analysis. These weak, unreplicated associations are hypothesis-generating and do not establish a general inverse stemness program.

### 3.7. S-Palmitoylation and Single-Cell Contextualization

UniProt-annotation-based topology screening evaluated the candidate proteins using an exploratory ±5-residue juxtamembrane positional rule (Supplementary Figure S7; Supplementary Table S12). Topology-filtered candidate cysteine residues were noted for AQP8 (Cys8 and Cys247) and TMIGD1 (Cys228 and Cys236). No residue met the stated criterion for BEST4, and no candidate was identified for the soluble or secreted proteins CA7, GUCA2A, and GUCA2B. These positions are not validated S-palmitoylation predictions and require experimental testing. OTOP2 was not retained in the final topology audit because its membrane orientation and residue-level provenance could not be verified from the archived analysis materials; no topology-based modification claim is made for OTOP2. Literature-curated cross-referencing with published colorectal and colonic single-cell RNA-seq atlases [16,17,18] suggested cell-type-specific expression patterns for the six genes represented in Supplementary Figure S8: AQP8 and CA7 in absorptive enterocytes, BEST4 in the specialized BEST4-positive epithelial population, GUCA2A/2B in goblet/enterocyte cells, and TMIGD1 in absorptive epithelial contexts. OTOP2 was not represented in the curated panel. Because no de novo quantitative single-cell reanalysis was performed, these annotations are contextual and absence from immune or stromal compartments cannot be concluded. Human Protein Atlas immunohistochemistry cross-referencing showed concordant protein-level downregulation in CRC tissue for the six genes assessed in the HPA analysis (AQP8, CA7, GUCA2A, GUCA2B, BEST4, and TMIGD1; Supplementary Table S13). OTOP2 was not included in this protein-level assessment because a comparable HPA immunohistochemistry entry was not available.

## 4. Discussion

This study selected seven candidate genes (AQP8, CA7, GUCA2A, GUCA2B, BEST4, TMIGD1, OTOP2) as highly accurate diagnostic biomarker candidates for CRC through integrated multi-dimensional bioinformatics characterization. The high AUC values in the discovery cohort and for five genes in GSE9348 demonstrate strong tissue discrimination within the analysed retrospective datasets; they do not validate a locked clinical test. This performance is consistent with these genes representing differentiated colonocyte markers whose expression is reduced during malignant transformation.

A central finding of this study is the convergent biological narrative that emerges from our multi-dimensional analysis: the candidate genes are predominantly expressed in differentiated colonic epithelial cells, while the six genes evaluated for stemness showed inverse trends and the survival analyses indicated primarily diagnostic rather than prognostic utility. This coherent pattern suggests that candidate-gene downregulation may reflect the loss of terminal differentiation associated with CRC initiation, as described in the classical adenoma-carcinoma sequence [19], rather than independently predicting disease progression.

AQP8 and TMIGD1 showed moderate positive correlations with the B-cell marker CD19 (r = 0.414 and r = 0.432, respectively). These exploratory bulk-expression correlations should not be interpreted as direct evidence of B-cell infiltration, immune activity, treatment response, or suitability for immunotherapy. Validation using immune-deconvolution methods or orthogonal experimental approaches is required.

The mRNAsi analysis using the published OCLR-derived scores revealed weak nominal negative correlations for AQP8 (rho = −0.142, nominal *p* = 0.019) and GUCA2B (rho = −0.135, nominal *p* = 0.026); however, neither association remained significant after correction across the six evaluated genes (BH-adjusted *p* = 0.078). These weak nominal trends provide a hypothesis-generating context for the possibility that candidate-gene loss accompanies oncogenic dedifferentiation [8]. The modest effect sizes and lack of multiplicity-adjusted significance preclude mechanistic conclusions.

Among the individual candidates, AQP8 showed strong dataset-specific tissue discrimination and a weak nominal inverse mRNAsi correlation. AQP8 belongs to the aquaporin family and participates in water and hydrogen peroxide transport across epithelial membranes. The topology-filtered positions Cys8 and Cys247 generate an exploratory hypothesis for experimental testing; positional proximity to transmembrane boundaries does not demonstrate S-palmitoylation, and evidence from other aquaporin family members cannot validate AQP8 modification [20]. For BEST4 (Q8NFU0), none of the five cysteine residues met the predefined ±5-residue juxtamembrane criterion; this result does not experimentally exclude palmitoylation.

The non-significant exploratory prognostic model (*p* = 0.639, AUC = 0.503) did not demonstrate prognostic discrimination in this cohort. This null result neither excludes prognostic effects nor establishes a role in tumour initiation rather than progression [21]. Future prognostic modeling should incorporate these genes alongside proliferative and microenvironment signatures for a more comprehensive risk stratification.

Current clinical CRC biomarkers include carcinoembryonic antigen (CEA) and carbohydrate antigen 19-9 (CA19-9), which are primarily used for postoperative monitoring and follow-up rather than early detection [22]. The blood-based methylated SEPT9 assay (Epi proColon) represents a clinically validated approach to non-invasive CRC screening, with performance approved for clinical use [23,24]. In contrast, the seven candidates identified in the present study are tissue-transcriptomic biomarkers derived from retrospective microarray analysis of resected CRC specimens. GUCA2A, in particular, has been independently identified as a CRC molecular marker in a separate bioinformatics study that also utilized the GSE44076 dataset [25], and the GUCA2A–GUCY2C tumour suppressor axis has been reported to be silenced across CRC subtypes in tissue samples [26], providing independent biological support for its relevance. The novelty of the present study lies not in proposing clinically superior biomarkers but in the integrated multi-dimensional evaluation—combining diagnostic accuracy, pathway enrichment, immune-marker correlations, stemness associations, post-translational modification assessment, and single-cell contextualization—within a single analytical framework. These candidates require experimental validation in prospective clinical specimens before any comparison with established clinical biomarkers can be meaningful.

The AUC values of 0.986–1.000 reported in this study were obtained exclusively from retrospective, curated tissue microarray datasets (GSE44076 and GSE9348). These values represent dataset-specific estimates of discriminative performance between CRC tissue samples and matched or unmatched normal colonic mucosa from the same cohorts; they should not be interpreted as established prospective clinical performance benchmarks. Translation to a prospective diagnostic setting would be affected by multiple factors not represented in these datasets: population heterogeneity across geographic, demographic, and comorbidity strata; variability in tumour stage distribution; the inclusion of benign colorectal conditions (e.g., diverticular disease, adenomas) and inflammatory conditions (e.g., inflammatory bowel disease) that may overlap with CRC expression profiles; pre-analytical variability in tissue procurement and RNA preservation; inter-laboratory platform and batch effects; and fundamental differences between tissue biopsy-based assays and non-invasive liquid biopsy approaches. Additionally, GSE9348 included only 12 normal tissue samples, limiting the statistical precision of the validation AUC estimates. These dataset-specific discrimination values are therefore reported as preliminary computational evidence for prioritising these candidates in subsequent experimental validation studies, and not as predictors of prospective clinical diagnostic performance.

### Limitations

This study has several important limitations. First, all analyses are computational; experimental validation through qRT-PCR, immunohistochemistry, and functional assays is essential to confirm diagnostic utility in clinical specimens. Second, single-cell expression patterns were derived through literature curation from published atlases rather than de novo scRNA-seq analysis; while this provides robust contextual support from large-scale published studies, quantitative single-cell validation in matched tumour-normal pairs is needed. Third, the topology-filtered cysteine assessment is an exploratory positional heuristic rather than a validated S-palmitoylation predictor; the candidate positions require experimental confirmation. Fourth, the prognostic model requires external validation in independent cohorts. Fifth, all training and validation cohorts derive from microarray platforms; RNA-seq-based validation would strengthen generalizability. The retention of BEST4 despite its position immediately outside the top-50 threshold represents an exploratory exception and may introduce selection bias; therefore, its diagnostic relevance requires further prospective validation. Despite these limitations, the convergence of several exploratory and partly overlapping analytical dimensions provides hypothesis-generating evidence supporting these candidate genes as priority candidates for translational CRC biomarker development. The concordant protein-expression findings obtained through Human Protein Atlas cross-referencing provide an additional supporting analytical dimension, interpreted with reference to the tissue-based Human Protein Atlas resource [27]. Furthermore, despite the use of an independent validation cohort, residual model-selection optimism and overfitting cannot be excluded, as candidate selection was performed entirely within the discovery dataset and both cohorts derive from retrospective microarray studies. Not all seven candidates were fully evaluated across platforms and secondary analyses: TMIGD1 was absent from the GPL570 array; OTOP2 did not reach significance in GSE9348 and was not included in the exploratory survival, immune-marker, mRNAsi, or HPA analyses. Consequently, no conclusions from those secondary analyses are extended to OTOP2. No locked multigene classifier with a prespecified cutoff was tested in an independent cohort. Future studies should employ larger, geographically and demographically diverse, multicentre cohorts; RNA-seq-based quantification; and prospective designs that include benign colorectal conditions, inflammatory bowel disease, premalignant lesions, and early-stage CRC to rigorously establish the clinical discriminative performance of these candidates.

## 5. Conclusions

We prioritized seven discovery-stage candidate genes with high retrospective tissue-discrimination AUCs; five showed similarly high discrimination in GSE9348 through integrated multi-dimensional bioinformatics analysis. The convergent characterization—cell-type-specific differentiated colonocyte expression, weak nominal stemness trends and descriptive immune-marker co-expression among the six evaluated genes, and exploratory topology-filtered candidate cysteine positions—provides a biological framework supporting their prioritization for further validation. These findings identify tissue-transcriptomic candidates with strong dataset-specific discriminative performance, warranting further experimental validation and prospective clinical evaluation in diverse cohorts before consideration for diagnostic application.

## Data Availability

The source datasets are publicly available from NCBI GEO (GSE44076 and GSE9348) and TCGA-COAD/UCSC Xena. A reproducibility code package containing scripts for candidate-selection reporting, ROC analysis, multiplicity correction, survival modelling, and topology auditing is provided with the revised submission. Because the original sample-level intermediate matrices were not included in the archived manuscript files, newly computed numerical results must be regenerated from the cited public datasets before being treated as confirmatory.

## Supplementary Materials

The following supporting information can be downloaded at: https://www.mdpi.com/article/10.3390/cimb48090916/s1.
