# Supplementary material for: Integrated Bioinformatics Identification and Multi-Dimensional Characterization of Diagnostic Hub Genes in Colorectal Cancer
Source: Curr Issues Mol Biol. 2026 Sep 8;48(9):916. doi: 10.3390/cimb48090916 (PMC13605869; doi:10.3390/cimb48090916)
Supplement: Supplementary file 1 [file cimb-48-00916-s001.zip › Supplementary_Tables.pdf]

Supplementary Tables

Integrated Bioinformatics Identification and Multi-Dimensional Characterization of Diagnostic Hub Genes in Colorectal Cancer

Zeynep Mutlu Coskun, Muhammet Coskun, Saime Sezer Sondas

Supplementary Table S1. Characteristics of GEO Datasets Used in This Study

Dataset	Platform	Samples (Normal/Tumor)	Purpose	Source
GSE44076	Affymetrix Human Genome U219 Array (HG-U219; GPL13667)	148 / 98	Discovery (DEG + STRING PPI analysis)	GEO
GSE9348	Affymetrix Human Genome U133 Plus 2.0 Array (GPL570)	12 / 70	External Validation	GEO
TCGA-COAD	Illumina HiSeq RNA-Seq	41 / 473	Survival, Stemness	UCSC Xena

GSE44076 comprises a total of 148 normal samples (98 matched adjacent-normal mucosa samples and 50 healthy normal colon samples) and 98 CRC samples. The primary paired differential-expression analysis used only the 98 CRC tissues paired with the 98 matched adjacent-normal samples.

Of the 473 TCGA-COAD tumour expression samples, 435 had available follow-up data and were used for survival analyses; 274 had complete expression and clinical records and were used for the elastic-net prognostic model and mRNAsi correlation analyses.

Supplementary Table S2. Summary of Differentially Expressed Genes (GSE44076)

Category	Count
Total Significant Genes	2,389
Upregulated	1,058
Downregulated	1,331
Threshold	$\lvert \log2FC \rvert > 1$ and BH-adjusted $P < 0.05$
Analysis	Paired limma, 98 CRC tumour vs 98 matched adjacent normal

The primary gene-level analysis identified 2,389 significant differentially expressed genes. A secondary probe-level sensitivity analysis identified 4,446 significant probes (2,093 upregulated and 2,353 downregulated); these probe counts should not be interpreted as unique genes.

Top 10 Upregulated Genes

Gene	log2FC	Adj. P-value
FOXQ1	5.66	1.92×10^{-51}
CLDN1	4.92	6.37×10^{-61}
DPEP1	4.80	4.08×10^{-39}
KIAA1199	4.70	3.31×10^{-60}
KRT23	4.44	2.44×10^{-29}
MMP7	4.42	5.60×10^{-37}
MMP3	4.27	9.30×10^{-31}
IL8	4.22	1.97×10^{-38}
TESC	3.95	1.29×10^{-38}
LY6G6D	3.84	3.80×10^{-26}

Top 10 Downregulated Genes

Gene	log2FC	Adj. P-value
CA1	−7.72	3.36×10^{-46}
CLCA4	−7.27	7.23×10^{-40}
TMIGD1	−6.93	2.76×10^{-51}
AQP8	−6.62	4.87×10^{-53}
MS4A12	−6.61	2.72×10^{-41}
GUCA2B	−6.55	4.76×10^{-55}
ZG16	−6.38	1.00×10^{-34}
CA4	−6.21	1.97×10^{-41}
GUCA2A	−6.05	5.15×10^{-45}
ADH1B	−5.81	2.96×10^{-47}

Multiple GPL13667 probes mapping to the same gene symbol were collapsed before model fitting by retaining the probe with the highest mean expression across all 246 samples. BH correction was subsequently performed across 20,033 genes.

Supplementary Table S3. MSigDB KEGG_LEGACY Over-Representation Analysis — Upregulated DEGs (Significant Pathways)

Pathway	P-value	Adj. P-value	Gene Count
Cell cycle	2.62×10^{-10}	6.09×10^{-8}	19
p53 signaling pathway	2.80×10^{-8}	3.25×10^{-6}	13
Wnt signaling pathway	4.41×10^{-6}	3.41×10^{-4}	16
IL-17 signaling pathway	2.27×10^{-5}	1.32×10^{-3}	11
Cellular senescence	1.54×10^{-4}	7.15×10^{-3}	13
Hippo signaling pathway	2.38×10^{-4}	8.53×10^{-3}	13
Protein digestion and absorption	2.57×10^{-4}	8.53×10^{-3}	10
ECM-receptor interaction	3.52×10^{-4}	1.02×10^{-2}	9
Bladder cancer	5.07×10^{-4}	1.31×10^{-2}	6
Colorectal cancer	1.39×10^{-3}	3.22×10^{-2}	8
Cushing syndrome	1.83×10^{-3}	3.85×10^{-2}	11

Supplementary Table S4. MSigDB KEGG_LEGACY Over-Representation Analysis — Downregulated DEGs (Significant Pathways)

Pathway	P-value	Adj. P-value	Gene Count
Drug metabolism	2.19×10^{-15}	3.25×10^{-13}	23
Retinol metabolism	2.80×10^{-15}	3.25×10^{-13}	19
Steroid hormone biosynthesis	5.22×10^{-15}	4.04×10^{-13}	18
Bile secretion	6.30×10^{-14}	3.66×10^{-12}	20
Pentose and glucuronate interconversions	7.34×10^{-13}	3.40×10^{-11}	13
Ascorbate and aldarate metabolism	3.00×10^{-12}	1.16×10^{-10}	12
Metabolism of xenobiotics by cytochrome P450	4.36×10^{-12}	1.44×10^{-10}	17
Porphyrin and chlorophyll metabolism	3.96×10^{-10}	1.15×10^{-8}	12
Chemical carcinogenesis	3.79×10^{-8}	9.78×10^{-7}	23
Nitrogen metabolism	4.62×10^{-5}	1.03×10^{-3}	5
Pancreatic secretion	4.90×10^{-5}	1.03×10^{-3}	11

Supplementary Table S5. Gene Set Enrichment Analysis (GSEA-KEGG, Top 20 Pathways)

Pathway	NES	NOM P	FDR q-val	Direction
RNA transport	1.983	0.000	0.000	Positively enriched
Ribosome biogenesis in eukaryotes	1.920	0.000	0.000	Positively enriched
Ribosome	1.747	0.000	0.002	Positively enriched

Pathway	NES	NOM P	FDR q-val	Direction
DNA replication	1.714	0.000	0.004	Positively enriched
Cell cycle	1.700	0.000	0.004	Positively enriched
Spliceosome	1.695	0.000	0.004	Positively enriched
RNA degradation	1.693	0.003	0.004	Positively enriched
Olfactory transduction	−1.817	0.000	0.000	Negatively enriched
Retinol metabolism	−1.814	0.000	0.000	Negatively enriched
Neuroactive ligand-receptor interaction	−1.810	0.000	0.000	Negatively enriched
Bile secretion	−1.776	0.000	0.001	Negatively enriched
Metabolism of xenobiotics by cytochrome P450	−1.754	0.000	0.001	Negatively enriched
Mineral absorption	−1.751	0.000	0.001	Negatively enriched
Aldosterone-regulated sodium reabsorption	−1.723	0.000	0.003	Negatively enriched
cAMP signaling pathway	−1.722	0.000	0.002	Negatively enriched
Glycolysis / Gluconeogenesis	−1.720	0.000	0.002	Negatively enriched
Pyruvate metabolism	−1.718	0.000	0.002	Negatively enriched
Regulation of lipolysis in adipocytes	−1.714	0.000	0.002	Negatively enriched
ABC transporters	−1.680	0.000	0.005	Negatively enriched
Intestinal immune network for IgA production	−1.674	0.000	0.006	Negatively enriched

NES: Normalized Enrichment Score. Positive NES indicates enrichment in CRC tumour; negative NES indicates enrichment in matched adjacent-normal tissue.

Supplementary Table S6. Dataset-Specific Tissue Discrimination of Candidate Genes: AUC Results

Gene	GSE44076 AUC	GSE9348 AUC
AQP8	0.993	0.999
CA7	0.994	1.000
GUCA2A	0.994	1.000
GUCA2B	0.995	1.000
BEST4	0.986	1.000
TMIGD1	0.995	N/A (not on GPL570)
OTOP2	0.995	Not evaluated

Note: AUC values derived from ROC analysis of CRC vs. normal tissue. GSE44076: 98 CRC vs. 98 normal; GSE9348: 70 CRC vs. 12 normal. TMIGD1 probe not present on GPL570 platform (GSE9348). OTOP2 was not included in the validation ROC analysis because its differential expression in GSE9348 was not statistically significant ($log_2FC = +0.50$, $P = 0.094$).

Supplementary Table S7. External Validation in GSE9348 Dataset

Gene	log2FC	P-value	Adj. P-value	Direction	Significant
AQP8	−8.40	1.17×10^{-20}	1.76×10^{-20}	Down	Yes
GUCA2B	−8.91	1.35×10^{-32}	8.08×10^{-32}	Down	Yes
GUCA2A	−6.66	6.94×10^{-23}	2.08×10^{-22}	Down	Yes
BEST4	−4.76	—	—	Down	P values not available
CA7	−3.76	2.85×10^{-17}	3.42×10^{-17}	Down	Yes
TMIGD1	—	—	—	N/A (not on platform)	—
OTOP2	+0.50	0.094	—	Up	No

Supplementary Table S8. Overall Survival Analysis of Six Evaluated Candidate Genes (GEPIA2, TCGA-COAD)

Gene	Log-rank P	Hazard Ratio (95% CI)	Median OS High (months)	Median OS Low (months)	Significant
AQP8	0.022	0.57 (0.35–0.93)	NR	82.3	Yes
CA7	0.910	1.00 (0.62–1.61)	NR	NR	No
GUCA2A	0.660	0.90 (0.56–1.45)	NR	NR	No
GUCA2B	0.510	0.85 (0.53–1.37)	NR	NR	No
BEST4	0.480	1.20 (0.74–1.94)	NR	NR	No
TMIGD1	0.410	0.82 (0.51–1.32)	NR	NR	No

NR: Not Reached. OTOP2 was not evaluated in this secondary survival analysis.

Supplementary Table S9. Six-Gene Exploratory Cox Proportional Hazards Prognostic Model

A. Model Coefficients

Gene	Coefficient (β)	Hazard Ratio	95% CI
AQP8	0.312	1.366	0.92–2.03
CA7	−0.160	0.852	0.58–1.25
GUCA2A	−0.695	0.499	0.31–0.80
GUCA2B	−0.247	0.781	0.52–1.17
BEST4	0.261	1.298	0.87–1.94
TMIGD1	0.316	1.372	0.93–2.02

B. Model Performance

Metric	Value
Patients (n)	274
Events (deaths)	66
Log-rank P (high vs. low risk)	0.639
Cross-validated AUC	0.503
Risk score formula	0.312×AQP8 − 0.160×CA7 − 0.695×GUCA2A − 0.247×GUCA2B + 0.261×BEST4 + 0.316×TMIGD1

Note: The six-gene prognostic model did not achieve statistical significance (P = 0.639). OTOP2 was not included in this secondary model; therefore, the result applies only to the six evaluated genes and is discussed as a limitation.

Supplementary Table S10. Correlations between Six Evaluated Candidate Genes and Selected Immune-Marker Genes (Spearman r, GSE44076 CRC Samples)

Gene	CD8+ T	CD4+ T	Treg	B cell	NK	Macrophage	PD-L1	IDO1	CTLA4
AQP8	0.259	0.088	0.078	0.414	0.130	−0.006	0.189	−0.025	−0.008
CA7	0.024	−0.138	−0.173	0.157	−0.076	−0.064	0.004	−0.114	−0.112
GUCA2A	0.177	−0.139	−0.186	0.275	0.066	−0.069	0.095	−0.006	−0.014
GUCA2B	0.096	−0.106	−0.239	0.253	−0.038	−0.106	−0.008	−0.048	−0.126
BEST4	−0.131	−0.086	−0.145	0.018	−0.204	0.071	0.002	−0.117	−0.055

Gene	CD8+ T	CD4+ T	Treg	B cell	NK	Macrophage	PD-L1	IDO1	CTLA4
TMIGD1	0.259	−0.022	−0.152	0.432	0.076	0.143	0.042	0.150	0.093

Coefficients are presented descriptively; raw P values were not preserved in the archived supplementary table and no significance claim is made. Spearman rank correlation between candidate-gene expression and selected immune-marker genes in CRC tumour samples from GSE44076 (n=98). These correlations reflect bulk mRNA co-expression and do not directly measure immune-cell abundance, infiltration, or functional activity. Immune markers used as proxies: CD8A (CD8+ T cells), CD4 (CD4+ T cells), FOXP3 (Tregs), CD19 (B cells), NCAM1 (NK cells), CD68 (macrophages), CD274 (PD-L1), IDO1, CTLA4. OTOP2 was not evaluated, and no immune-association inference is made for OTOP2.

Supplementary Table S11. mRNA Stemness Index (mRNAsi) Correlations for Six Evaluated Candidate Genes

Gene	Spearman ρ	P-value	BH-adjusted P	Interpretation
AQP8	−0.142	0.019	0.078	Weak nominal inverse correlation; not significant after BH correction
CA7	−0.001	0.986	0.986	No meaningful correlation
GUCA2A	−0.074	0.223	0.446	Non-significant exploratory trend
GUCA2B	−0.135	0.026	0.078	Weak nominal inverse correlation; not significant after BH correction
BEST4	−0.057	0.345	0.517	Non-significant exploratory trend
TMIGD1	−0.042	0.491	0.589	No meaningful correlation

Data source: Malta et al. (2018) OCLR-based mRNAsi scores from UCSC Xena; n = 274 TCGA-COAD patients. OTOP2 was not evaluated in this secondary analysis.

Supplementary Table S12. Topology-Filtered Candidate Cysteines (Exploratory Positional Heuristic)

Gene	UniProt ID	Length (aa)	TM Domains	Total Cys	Candidate Sites	Status	Rationale
AQP8	O94778	261	6	6	Cys8, Cys247	Exploratory	Cys8 (cytoplasmic N-terminus) and Cys247 (C-terminus near TM6) are topology-filtered candidate residues; AQP4 evidence does not validate AQP8 modification.

Gene	UniProt ID	Length (aa)	TM Domains	Total Cys	Candidate Sites	Status	Rationale
TMIGD1	Q6UXZ0	262	1	8	Cys228, Cys236	Exploratory	Cys228 and Cys236 are juxtamembrane cysteines in the cytoplasmic tail. Their positions were selected by the exploratory distance rule and require experimental validation.
CA7	P43166	264	0	4	None predicted	Not identified	Soluble cytoplasmic protein without membrane association.
GUCA2A	Q02747	115	0	7	None predicted	Not identified	Secreted peptide; all cysteines form disulfide bonds.
GUCA2B	Q16661	112	0	7	None predicted	Not identified	Secreted peptide; all cysteines form disulfide bonds.
BEST4	Q8NFU0	473	4	5	None under ±5-aa criterion	Exploratory	No cytoplasmic cysteine lies within ±5 residues of a UniProt-annotated transmembrane boundary; this topology-based result does not experimentally exclude palmitoylation.
OTOP2	Q7RTS6	562	12	16	Cys87, Cys161, Cys492	Exploratory	Cys87 (+5 residues from TM2 end at position 82), Cys161 (+4 residues from TM4 end at position 157), and Cys492 (−3 residues from TM11 start at position 495); orientation inferred from OTOP family structural data, not explicitly annotated in UniProt Q7RTS6; no palmitoylation annotations present in UniProt entry.

Analysis based on UniProt topology annotations. The ±5-residue rule is an exploratory positional heuristic, not a validated S-palmitoylation predictor; experimental validation is required.

Supplementary Table S13. Human Protein Atlas (HPA) Immunohistochemistry Validation

Gene	Normal Colon Expression	CRC Expression	Staining Pattern	Antibody	Consistency with mRNA
AQP8	High (cytoplasmic/membrane)	Not detected	Membrane, apical	HPA046259	Consistent ↓
CA7	Medium (cytoplasmic)	Low/Not detected	Cytoplasmic	HPA005993	Consistent ↓
GUCA2A	High (cytoplasmic)	Not detected	Cytoplasmic, granular	HPA017516	Consistent ↓
GUCA2B	Medium-High	Low/Not detected	Cytoplasmic	HPA014844	Consistent ↓
BEST4	Medium (apical membrane)	Not detected	Membrane	HPA058791	Consistent ↓
TMIGD1	Medium (membrane)	Low/Not detected	Membrane	HPA042592	Consistent ↓

Data source: Human Protein Atlas v23.0 (proteinatlas.org). All six genes assessed in the HPA analysis showed reduced protein expression in CRC tissue compared with normal colon, consistent with the transcriptomic findings. OTOP2 was not evaluated because a comparable HPA immunohistochemistry entry was not available for this analysis.
